# Innovative Dual Catheter Irrigation Technique for Clot Retention in a Hospice Setting: A Case Report

**DOI:** 10.1002/ccr3.70849

**Published:** 2025-09-04

**Authors:** Katie Bland, David Wenzel, Luke Feathers

**Affiliations:** ^1^ Palliative Care LOROS Hospice Leicester UK; ^2^ Population Health Sciences University of Leicester Leicester UK

**Keywords:** clot retention, hospice‐based intervention, irrigation, palliative care, suprapubic catheterisation

## Abstract

Clot retention can be challenging to manage in a hospice setting in the final days, especially if a blocked urethral catheter cannot be replaced. Small‐bore suprapubic irrigation using equipment commonly available in hospices can provide symptom relief without the need to transfer to acute settings.

## 1 Introduction

Acute gross haematuria is a common occurrence in hospice settings, often secondary to malignancies of the urinary tract and complicated by anatomical distortion. The accumulation of blood clots can obstruct urinary outflow, constituting a urological emergency that necessitates prompt intervention to minimize pain and distress at the end of life [1]. Standard management typically involves the insertion of a three‐way catheter with manual and continuous bladder irrigation [2]. When these measures fail, definitive hemostasis via specialist urological procedures is often required, which usually necessitates transfer to an acute care facility [3].

We present a case demonstrating the management of clot retention within a hospice environment using an innovative dual catheter irrigation technique. This approach honored the patient's wish to remain within his preferred care setting and avoided hospital readmission.

## 2 Case History

A 75‐year‐old male with a neuroendocrine prostatic carcinoma was admitted to our hospice for symptom control following palliative radiotherapy. During his admission, he developed severe macroscopic haematuria, resulting in acute urinary retention. Standard hospice practice of inserting a urethral three‐way catheter for manual irrigation was attempted but was unsuccessful; neither a three‐way catheter nor a Foley catheter could be placed. Consequently, the patient was transferred to a urology unit.

**FIGURE 1:**
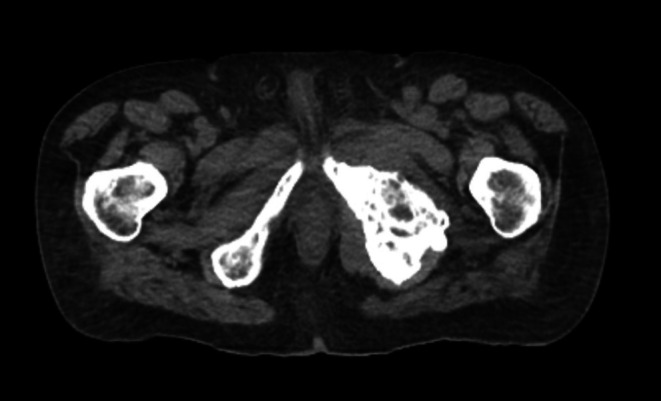
Pre admission CT scan demonstrating pelvic structural abnormality.

In the acute hospital setting, despite the use of cystoscopy, the urology team was unable to place a three‐way catheter due to significant anatomical distortion. A representative axial CT image obtained during preadmission radiotherapy planning is shown in Figure 1. This image demonstrates extensive pelvic anatomical distortion secondary to tumor burden. A 14Fr Foley catheter was inserted instead, and the patient was returned to the hospice.

Within 24 h of readmission, the Foley catheter became obstructed and could not be cleared. Attempts at flushing with sterile water were only partially successful; fluid could be instilled with difficulty but could not be aspirated, leading to the introduction of an additional 100 mL of fluid into the bladder without effective drainage. This exacerbated the patient's symptoms of pain and bladder spasms.

Readmission to the urology department for further intervention was discussed. However, due to his prior traumatic experience and ineffective symptom control during hospitalization, the patient declined. He expressed a clear preference for symptomatic management within the hospice, even if this approach potentially shortened his life.

## 3 Treatment

Pharmacological management was optimized, including a continuous subcutaneous infusion of midazolam (5 mg/24 h) and hyoscine butylbromide (120 mg/24 h), alongside oral methadone 15 mg twice daily. Despite these measures, he required additional oral oxycodone (totalling 80 mg in 20 mg PRN doses) for symptom control. In light of ongoing distress and following comprehensive discussion regarding risks and benefits, a suprapubic catheter was placed using a Rocket Safety Drain (R58800‐08‐SD)—an off‐label use of a device more commonly employed for ascitic drainage.

An ultrasound was performed just cranial to the pubic bone to confirm bladder location and ensure no significant anatomical distortion. A skin bleb was raised with 8 mL of 1% lidocaine, followed by a small incision. The Rocket Safety Drain was then advanced through the incision and into the bladder. Once intravesical placement was confirmed, the introducer needle was withdrawn, and the drain was secured using Tegaderm dressings.

In contrast to the traumatic urethral catheterization, the patient tolerated the procedure exceptionally well and was able to fall asleep during the process.

Once in place, the suprapubic catheter immediately relieved the urinary retention. The existing blocked urethral catheter was left in situ. Using the urethral catheter, 1.5 L of sterile water was instilled into the bladder with a 50 mL Luer Lock syringe. A push‐pull technique was employed—repeatedly injecting fluid into the bladder via the urethral catheter and allowing passive drainage through the suprapubic catheter. This generated hydraulic turbulence within the bladder, mobilizing and ultimately dislodging the obstructive clot. Once both catheters were draining freely, continuous irrigation was established via the suprapubic catheter to maintain patency.

## 4 Outcome

Following successful clearance of the blockage, both the urethral and suprapubic catheters were left in situ and maintained on a three‐way irrigation system. Irrigation fluid was infused via the suprapubic catheter and drained via the urethral catheter. Although intermittent catheter blockages occurred, these were consistently managed through manual flushing, and blockages were minimized by increasing the infusion rate.

A contingency plan for potential catheter failure was not formally established; decisions regarding catheter management would have been based on clinical judgment, prognosis, and patient preference at the time of complication.

The patient's haematuria persisted and necessitated ongoing irrigation; however, his symptom control remained excellent, requiring minimal additional analgesia or sedation. An infusion of hyoscine butylbromide was maintained to mitigate bladder spasms secondary to intermittent catheter blockage.

The patient's condition continued to decline in keeping with his underlying disease trajectory, and he died peacefully 10 days after suprapubic catheter placement. Importantly, no complications related to the suprapubic catheterization were observed, and the patient remained within his preferred care setting until the end of life.

## 5 Discussion

Clot retention is a recognized urological emergency that presents unique challenges when encountered within hospice settings, where resources and specialist support may be limited. Standard management typically includes the placement of a three‐way urethral catheter with manual and continuous bladder irrigation to evacuate clots and maintain urinary drainage [2]. In cases where conventional catheterization fails, surgical or endoscopic intervention is often required [3]. However, in palliative care environments, such invasive approaches may conflict with patients' goals of care, emphasizing the need for symptom‐focused, minimally invasive strategies.

In this case, a dual catheter irrigation system utilizing a urethral Foley catheter and a suprapubic Rocket Safety Drain was employed successfully to manage refractory clot retention. Notably, there is limited existing literature describing the off‐label use of central venous catheters or other small‐bore drains for suprapubic urinary diversion. Bilehjani and Fakhari (2017) reported the successful use of central venous catheters for suprapubic catheterization in cardiac surgical patients, highlighting minimal complications and ease of insertion [4].

The placement of a suprapubic drain in the hospice setting provided immediate symptom relief and allowed ongoing bladder irrigation without necessitating transfer to an acute care facility. Importantly, informed consent was obtained, and the risks of infection, bleeding, and off‐label device use were explicitly discussed with the patient. The benefits, including preservation of comfort, maintenance of consciousness, and respect for place‐of‐care preferences, outweighed the potential risks in this context.

Alternative chemical methods for clot dissolution have also been described. Xu et al. (2020) reported the use of dilute hydrogen peroxide bladder irrigation (3%) to assist in the mechanical disruption of bladder clots in cases of urinary retention refractory to standard manual evacuation [5]. However, the hydrogen peroxide was an addition to standard irrigation fluid, rather than infused into the bladder and left. This approach carries potential risks of mucosal irritation and bladder wall injury, which would be more likely with the prolonged exposure of bladder irrigation without drainage.

Other studies have demonstrated clot resolution using instillations of streptokinase [6], alteplase [7] and chymotrypsin [8] with reasonable success. However, all relied on alternate methods of clot evacuation in the event of failure and necessitated the availability of specific medications to lyse clots.

In the context of our patient, given the available resources, the urgency of symptom relief, and the desire to avoid additional risk, mechanical irrigation via a dual catheter system was preferred over chemical lysis strategies.

Management of the dual catheter system, however, was labor‐intensive and required continuous clinical vigilance. Intermittent blockage of either catheter necessitated troubleshooting and occasional reversal of irrigation flow, underscoring the importance of adaptability and clinical judgment in such palliative interventions. Previous studies have emphasized that catheter‐related complications such as blockage are frequent even in standard practice, with occlusion rates varying between 5% and 10% in long‐term catheter use [9]. Therefore, familiarity with manual irrigation techniques and flexible problem‐solving approaches are essential skills for palliative care teams undertaking similar interventions.

The absence of significant complications and the reported satisfaction of both patient and family reflect the importance of individualized, preference‐based care in the hospice environment. This case further supports the broader principle that even complex urological emergencies can be managed effectively within palliative care settings when conventional options are unavailable or undesirable.

In palliative care, authentic patient choice is often challenged by biological emergencies that demand urgent intervention. Sociological critiques have argued that end of life “choices” can be constrained or shaped by institutional structures and medical imperatives, rather than reflecting true autonomy [10]. In this case, the team consciously overcame the biological imperative to surgically evacuate clots or transfer to an acute care setting, instead facilitating an individualized, preference‐driven management plan within the hospice. This highlights the critical role of clinical creativity and flexibility in upholding patient autonomy during terminal care.

Further research into minimally invasive urinary drainage techniques tailored for palliative care populations is warranted. Formal protocols for off‐license suprapubic catheterization approaches, especially using small‐bore devices, could help improve access to symptom control strategies for patients in similar circumstances.

This case highlights the feasibility and effectiveness of an innovative, dual catheter irrigation approach to managing refractory clot retention within a hospice setting. The use of a Rocket Safety Drain as a suprapubic catheter provided immediate symptomatic relief, preserved patient dignity, and honored end of life care preferences without resorting to hospital readmission.

Our experience demonstrates that with appropriate informed consent and clinical oversight, unconventional yet patient‐centered interventions can significantly improve quality of life at the end of life. Palliative care teams should be aware of alternative catheterization techniques and be prepared to adapt standard urological practices to meet the unique needs of their patients.

Ultimately, shared decision‐making, flexibility, and symptom‐focused innovation remain essential pillars in the palliative management of urological emergencies.

## Author Contributions

**Katie Bland:** conceptualization, formal analysis, investigation, writing – original draft. **David Wenzel:** conceptualization, data curation, formal analysis, formal analysis, writing – original draft, writing – original draft, writing – review and editing, writing – review and editing. **Luke Feathers:** conceptualization, project administration, supervision, writing – review and editing.

## Consent

The patient verbally consented to publication of a case report before his death. Formal, written consent was obtained from his next of kin after death.

## Conflicts of Interest

The authors declare no conflicts of interest. Katie Bland's post is funded by Health Education England while Luke Feathers is medical director of LOROS Hospice. David Wenzel is funded by a Wellcome Trust Grant via the Mental Health and Neurosciences Doctoral Training Programme (grant number MHN DTP—223508/Z/21/Z). Funders had no involvement in the production or conceptualisation of this work.

## Data Availability

All relevant clinical information is included within the manuscript. No additional datasets were generated or analyzed during the preparation of this case report. Should the editorial or review team require further details, Supporting Information can be provided by the corresponding author upon reasonable request.
